# Early spatial attention deployment toward and away from aggressive voices

**DOI:** 10.1093/scan/nsy100

**Published:** 2018-11-09

**Authors:** Nicolas Burra, Dirk Kerzel, David Munoz Tord, Didier Grandjean, Leonardo Ceravolo

**Affiliations:** 1 Faculté de Psychologie et des Sciences de l’Education, University of Geneva, Geneva, Switzerland; 2 Neuroscience of Emotion and Affective Dynamics Lab, University of Geneva, Geneva, Switzerland; 3 Swiss Center for Affective Sciences, University of Geneva, Geneva, Swizerland

**Keywords:** LPCpc, N2ac, spatial attention, threat

## Abstract

Salient vocalizations, especially aggressive voices, are believed to attract attention due to an automatic threat detection system. However, studies assessing the temporal dynamics of auditory spatial attention to aggressive voices are missing. Using event-related potential markers of auditory spatial attention (N2ac and LPCpc), we show that attentional processing of threatening vocal signals is enhanced at two different stages of auditory processing. As early as 200 ms post-stimulus onset, attentional orienting/engagement is enhanced for threatening as compared to happy vocal signals. Subsequently, as early as 400 ms post-stimulus onset, the reorienting of auditory attention to the center of the screen (or disengagement from the target) is enhanced. This latter effect is consistent with the need to optimize perception by balancing the intake of stimulation from left and right auditory space. Our results extend the scope of theories from the visual to the auditory modality by showing that threatening stimuli also bias early spatial attention in the auditory modality. Attentional enhancement was only present in female and not in male participants.

## Introduction

Because the detection of threatening events is crucial for survival (LeDoux, 1996), our attentional system is believed to quickly and automatically engage in the evaluation of threatening events (Öhman and Mineka, 2001). In vision, facilitated attentional orienting to threat was often observed for angry faces (‘anger superiority effect’, for instance Hansen and Hansen, 1988 and Öhman *et al.*, 2010). Typically, participants are better at detecting angry as compared to happy faces, which was taken as evidence for the idea that threatening stimuli capture attention because a pre-attentive threat-detection system automatically guides visual attention to their location (‘threat capture hypothesis’, Öhman and Mineka, 2001). However, threat can also take the shape of an auditory signal. Indeed, vocal signals represent one of the most relevant sound categories (Belin *et al.*, 2004). Processing of threatening or aggressive vocal signals involves brain regions dedicated to voice perception, for instance the superior temporal sulcus (STS), the orbitofrontal and parietal cortices, as well as the amygdala (Sander *et al.*, 2005; Ceravolo *et al.*, 2016), a subcortical structure also involved in threat detection (LeDoux, 1996). In line with the threat capture hypothesis, detection of aggressive voices is rapid (Sauter and Eimer, 2010) and automatic (Sander *et al.*, 2005; Gädeke *et al.*, 2013). Interestingly, larger activation of STS occurs even when the focus of attention is directed away from the voice (Grandjean *et al.*, 2005), emphasizing the automatic nature of threat detection.

There is also evidence to suggest that threatening vocalizations affect early sensory stages of visual processing. Brosch *et al.* (2008a) demonstrated that detection of visual probes was faster at the location of threatening vocalizations. Using event-related potentials (ERPs), it was observed that faster detection was accompanied by a larger P1 component to probes shown at the location of an aggressive vocal signal, suggesting that sensory gain was enhanced (Brosch *et al.*, 2009). Even though these results suggest that aggressive vocal signals have an impact on early sensory stages of visual processing, the temporal dynamics underlying attentional deployment toward threatening voices remain largely unknown. Actually, effects of attention were measured to the probe, which appeared 500 ms after the aggressive vocal signal. Thus, the time course of attentional deployment to the threatening vocal signal itself remains unknown.

The main objective of the present paper was to provide a systematic investigation of the temporal dynamics of early spatial attention toward threatening vocal signals in the auditory modality. In the visual modality, a lateralized ERP, the N2pc, was previously used as an index of spatial attention (Luck and Hillyard, 1994; Eimer, 1996). The N2pc is a negative deflection of the ERP waveform at posterior electrodes PO7/8 contralateral to the selected stimulus (for a review, see Luck, 2012). It occurs between 180 and 300 ms after stimulus onset. Because the N2pc is calculated by subtracting ipsilateral from contralateral ERPs, it is relatively independent from overlapping non-lateralized ERP components. The N2pc component was shown to provide electrophysiological evidence for the anger superiority effect (enlarged N2pc ~200–300 ms for angry *vs* happy target faces; Feldmann-Wustefeld *et al.*, 2011; Weymar *et al.*, 2011; Burra *et al.*, 2016). In the auditory modality, the deployment of spatial attention was recently associated with a lateralized ERP component analogous to the N2pc. Gamble and Luck (2011) presented two lateralized stimuli through separate loudspeakers, and participants were required to detect the presence of the target. Presumably, spatial attention was oriented to the location of the target to discriminate it from the distractor on the other side. For this reason, a negative deflection contralateral to the attended stimulus at anterior central sites from 200 to 300 ms after stimulus onset was interpreted as a correlate of spatial attention in an auditory scene. Interestingly, the contralateral negativity, referred to as N2ac, was followed by a contralateral positivity at posterior sites, the LPCpc. The LPCpc is thought to reflect the reorienting of spatial attention to the center after target localization (Gamble and Luck, 2011; Gamble and Woldorff, 2015; Lewald *et al.*, 2016) and may therefore index attentional disengagement (Posner, 1980). Thus, the N2ac and LPCpc may reveal the dynamics of attentional orienting toward and away from threatening vocal signals.

Previous research measured the neural dynamics of vocal emotion processing (reviewed in Schirmer and Kotz, 2006, and Kotz and Paulmann, 2011) with an emphasis on threat. Using non-lateralized ERPs, the perception of vocal emotional expressions was shown to occur in three stages (Schirmer and Gunter, 2017): first, an early sensory stage involving acoustic feature analysis; second, the detection of emotional salience as derived from the integration of features; and third, a cognitive evaluation of the emotional significance of the voice. The N100 component presumably reflects early sensory analysis (i.e. processing low-level acoustic features; Hyde, 1997), whereas the integration of acoustic features related to emotions and the detection of emotional salience is thought to take place ~200 ms after stimulus onset (Paulmann and Kotz, 2008; Sauter and Eimer, 2010; Liu *et al.*, 2012; Pinheiro *et al.*, 2013; Schirmer *et al.*, 2013; Pell *et al.*, 2015). The integration persists even when the information is task-irrelevant (Wambacq *et al.*, 2004). Finally, later stages of emotional voice processing were associated with the extraction of emotional meaning. For instance, the P300 (Wambacq *et al.*, 2004; Thierry and Roberts, 2007; Campanella *et al.*, 2010; Liu *et al.*, 2012) and the late positive potential (Paulmann *et al.*, 2013; Pell *et al.*, 2015; Pinheiro *et al.*, 2016) revealed differentiation between emotional as compared with neutral auditory signal.

The above review of the literature confirms that despite the considerable progress in the understanding of vocal emotion processing, evidence for an early deployment of auditory attention toward the location of aggressive vocal signals is still missing. To fill this void, ERP markers of both spatial attention deployment (N2ac) and reorienting (LPCpc) were measured in a task involving the detection of voices expressing either aggressiveness or happiness. According to the threat capture hypothesis (Öhman and Mineka, 2001), we predicted more accurate and faster detection of threatening voices. However, we also expect an advantage for happy compared to neutral stimuli, since positive stimuli were also shown to capture attention in the visual (Brosch *et al.*, 2008b) and auditory modality (Pinheiro *et al.*, 2017). Regarding encephalography (EEG), we predicted an N2ac to all target stimuli (Gamble and Luck, 2011), but a larger N2ac to aggressive as compared to happy voices, similar to the enhanced N2pc to angry *vs* happy faces (Feldmann-Wustefeld *et al.*, 2011; Weymar *et al.*, 2011; Burra *et al.*, 2016). Moreover, we predicted that following early attentional orienting, threatening voices would also affect the later attentional reorienting, which is, in our case, the flip side of attentional disengagement (Posner, 1980).

## Materials and method

### Participants

There were 35 right-handed healthy participants (14 male; mean age, 20.7 ± 2.54; min–max, 18–32 years). Data from one male participant were discarded from the analyses because a likely psychiatric condition was revealed after testing. No statistical methods were used to predetermine sample size. However, our sample size was similar or larger than in related publications (Gamble and Luck, 2011; Gamble and Woldorff, 2015). All participants completed the Spielberger State/Trait Anxiety Inventory (STAI-S) and STAI-T questionnaires (Spielberger *et al.*, 1983).

### Stimuli

Stimuli were meaningless utterances taken from the Geneva Multimodal Expression Portrayals database (Bänziger and Scherer, 2007). We used stimuli produced by eight professional actors (four males and four females) who pronounced ‘Aah’ in either an aggressive, happy or a neutral voice, resulting in 24 different stimuli. The duration of vocalizations was shortened so that stimuli lasted ~700 ms but their emotional content was preserved (see Supplementary Material in Ceravolo *et al.*, 2016). Recently, it was suggested that low-level confounds might explain attentional capture by emotional content in the visual modality (for instance the presence of visible teeth in happy face stimuli, see Savage *et al.*, 2013). Similarly, a systematic imbalance in low-level features between emotional and neutral prosodies might explain behavioral or/and electrophysiological differences. To preclude this, the stimuli were adjusted and we confirmed that basic voice acoustics were comparable between neutral, happy and aggressive voices (cf. characteristics of the original stimuli in Supplementary Table 1 and adjusted stimuli in Supplementary Table 2).

However, preliminary analysis showed that accuracy was lower for aggressive as compared to happy voices. Detailed analyses revealed that one aggressive stimulus was only detected at chance level. In addition, this stimulus was assessed as very low in terms of valence, emotional intensity or threatening content. Therefore, we decided to remove it from all behavioral and EEG analyses. The summary characteristics of neutral, happy and aggressive stimuli were not changed by its removal (Table 1). Finally, an independent group of participants also assessed the threatening value of the remaining stimuli (see Supplementary data).

**Table 1:** Results of an analysis of pitch, duration and intensity for the 22 stimuli that remained after the emotional prosody of one of the original speakers was removed (i.e. two stimuli, see text). Means and s.d. of acoustic parameters for neutral, aggressive and happy vocal signals are shown together with the *F*- and *P*-values of a one-way ANOVA. Critically, the factor emotional expression (neutral, aggressive and happy) was not significant, showing that low-level stimulus characteristics did not differ as a function of emotion

	Pitch (Hz)	Duration (ms)	Intensity (dB)
Neutral (*n* = 8)	247 ± 70	549 ± 97	81.30 ± 2.63
Aggressive (*n* = 7)	302 ± 93	597 ± 12	79.70 ± 10.4
Happy (*n* = 7)	311 ± 54	592 ± 63	80.27 ± 1.44
*F*(2, 19)	1.69	1.13.	0.14
*P*	0.21	0.34	0.87

Hz, Hertz; ms, milliseconds; dB, decibels.

### Procedure

**Fig. 1:**
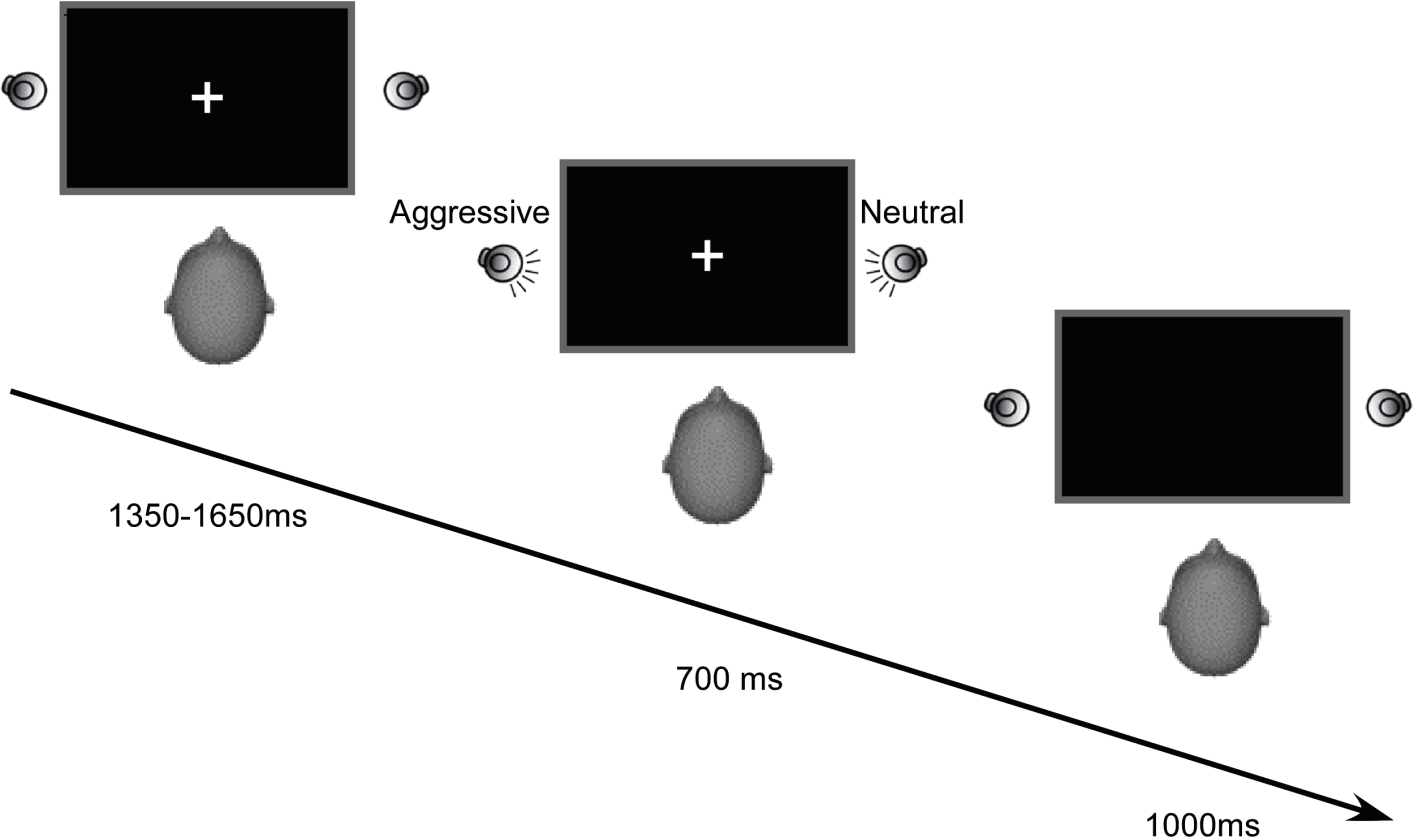
Illustration of one experimental trial. The trial started with the presentation of the fixation cross for 1350–1650 ms. Next, two vocal signals were presented through two lateral loudspeakers for a maximal duration of 700 ms. On target-present trials, one of the two signals was an emotional vocal signal (either aggressive or happy). On target-absent trials, the two voices were neutral. Participants indicated whether an emotional target voice was present or absent. Stimulus presentation was followed by a 1000 ms blank screen.

Our experiment took place in a soundproof cabin (Diatec AG, Switzerland). Participants sat 85 cm from a computer screen with loudspeakers (Logitech, LS11) located at approximately ±15 degrees of azimuth and 5 degrees of elevation relative to the participants’ head (Figure 1). The presentation of the stimuli and the collection of the responses were controlled by a computer running MATLAB 2009b (The Math Works, Natick, USA), the Psychtoolbox v.3 and a high-definition audio card (Realtek Inc., Hsinchu, Taiwan).

We instructed participants to keep their eyes on a 0.5 × 0.5 degrees fixation cross-presented in the center of the computer screen throughout the experiment. Each trial began with the presentation of the fixation cross for a randomly determined duration between 1350 and 1650 ms. On each trial, two sounds were presented through two loudspeakers for 700 ms. After the response, a blank screen appeared for 1000 ms. Stimulus intensity was 65 dB sound pressure level.

Participants were requested to indicate as accurately and rapidly as possible the presence or absence of an emotional target by pressing one of two keys on a regular keyboard with two fingers of the right hand. Key-to-response mapping was counterbalanced across participants. On target-present trials, an aggressive or happy target voice was presented together with a neutral voice. On target-absent trials, two neutral voices were presented. A target was present on half of the trials. An aggressive voice was presented on half of the target-present trials and a happy voice on the other half. Each of the 16 target stimuli was repeated 14 times so that each emotion (happy/aggressive) was presented on 224 trials. Target stimuli appeared equally likely in left and right auditory space. Overall, there were 896 trials per participant.

In two groups of participants, the aggressive and happy targets were either blocked (cf. Gamble and Luck, 2011) or randomly interleaved. Block order was counterbalanced. Blocking or randomly interleaving conditions changed target predictability (Burra and Kerzel, 2013). However, no behavioral differences emerged between the blocked and random group and group was therefore collapsed in the following analyses (see Supplementary data for analyses including stimulus order).

The experiment started with 1 block of 56 trials in which participants were familiarized with the paradigm. In the demonstration block, simple sine wave sounds of 600 ms were used instead of the emotional targets. Shortly before the experiment, all voice stimuli were played once in order to balance foreknowledge of the materials. Following the main task, participants were required to rate the material they heard during the experiment on a continuous scale (sound valence: −100 = negative to 100 = positive; emotional intensity or the amount of subjective emotional content: 0 = low to 100 = high emotional content). The order of stimulus presentation was random and both ratings were given sequentially. Finally, the level of anxiety was assessed.

### Behavioral analysis

To take into account within- and between-subject variance, we used the lmerTest (Kuznetsova *et al.*, 2014) and lme4 packages (Bates *et al.*, 2014) in R (Team, R.C., 2014) to perform a general linear mixed effect model on the reaction times of correct responses. Since the data were not normally distributed, the log of the reaction times was used instead of the raw values (see Supplementary Fig. 1 for an illustration). The log of the reaction times (for aggressive, happy and neutral voices) was the dependent variable while emotional expression (aggressive, happy and neutral) was introduced as a fixed effect and participant was introduced as random effect. We corrected *P*-values for multiple comparisons using Tukey’s method implemented in the glht (multcomp package; Genz *et al.*, 2008). For the sake of clarity, we report the raw data in millisecond in Supplementary Table 3. The effect of gender is reported in the Results section but the methods are reported in the Supplementary data.

### EEG recording and analysis

We used a Biosemi ActiveTwo system with electrode positions based on the International 10–10 system. We recorded from 32 Ag/AgCl scalp sites (Fp1/2, Fz, F3/4, F7/8, Cz, C3/4, T7/8, Pz, P1/2, P3/4, P5/6, P7/8, P9/10, POz, PO3/4, PO7/8, Oz, O1/2 and Iz). The left and right mastoid electrodes were used as offline references. Electrodes placed at the outer right and left canthi measured the horizontal electro-oculogram (HEOG) and electrodes above and below the right eye measured the vertical electro-oculogram (VEOG). BrainVision Analyzer 2.1 (BrainProduct, Products, Gilching, Germany) was used for offline analysis. The data were band-pass filtered using a zero phase-shift Butterworth filter with half-amplitude cut-offs at 0.1 and 40 Hz. The filter was set to 0.1 and 10 Hz for HOEG and VEOG. Then, the data were re-referenced to the average of the left and right mastoids (see Gamble and Woldorff, 2015) and we applied an independent component analysis, implemented in BrainVision Analyzer, to reduce the impact of eye blinks on the EEG signal (see Drisdelle *et al.*, 2017). Then, we applied a baseline correction of 100 ms and removed epochs with blinks (difference in VEOG >60 μV during a period of 150 ms), saccadic eye movement to the left or right (difference in HEOG >30 μV during a period of 150 ms) and bad epochs (any electrodes >80 μV). Further, trials with incorrect behavioral responses and with a response <200 ms or >2000 ms were excluded from the analysis (8%). Overall, 11.7% of data were removed, but this percentage did not differ between target conditions [12.2% for aggressive, 11.8% for happy and 11.3% for target-absent, *F*(2, 66) = 0.77, *P* = 0.46]. Finally, we collapsed waveforms across the different speakers to reduce physical stimulus confounds in the analyses and calculated the difference wave between the average contralateral and ipsilateral waveforms for target-present trials, separately for aggressive and happy voices.

We extracted the mean amplitude of the contralateral minus ipsilateral waveform during a time interval around the peak of the N2ac between 200 and 300 ms. For the LPCpc, a 400–600 ms window was used. The N2ac was extracted in a cluster of eight anterior electrode sites (C3/4, CP5/CP6, FC5/FC6 and T7/T8) and the LPCpc in a cluster of eight parietal electrodes (O1/O2, P7/P8, PO3/PO4 and P3/P4), comparable with the previous literature (Gamble and Luck, 2011; Gamble and Woldorff, 2015; Lewald *et al.*, 2016). To rule out effects of eye movements, we also analyzed the HEOG during these time windows.

**Fig. 2:**
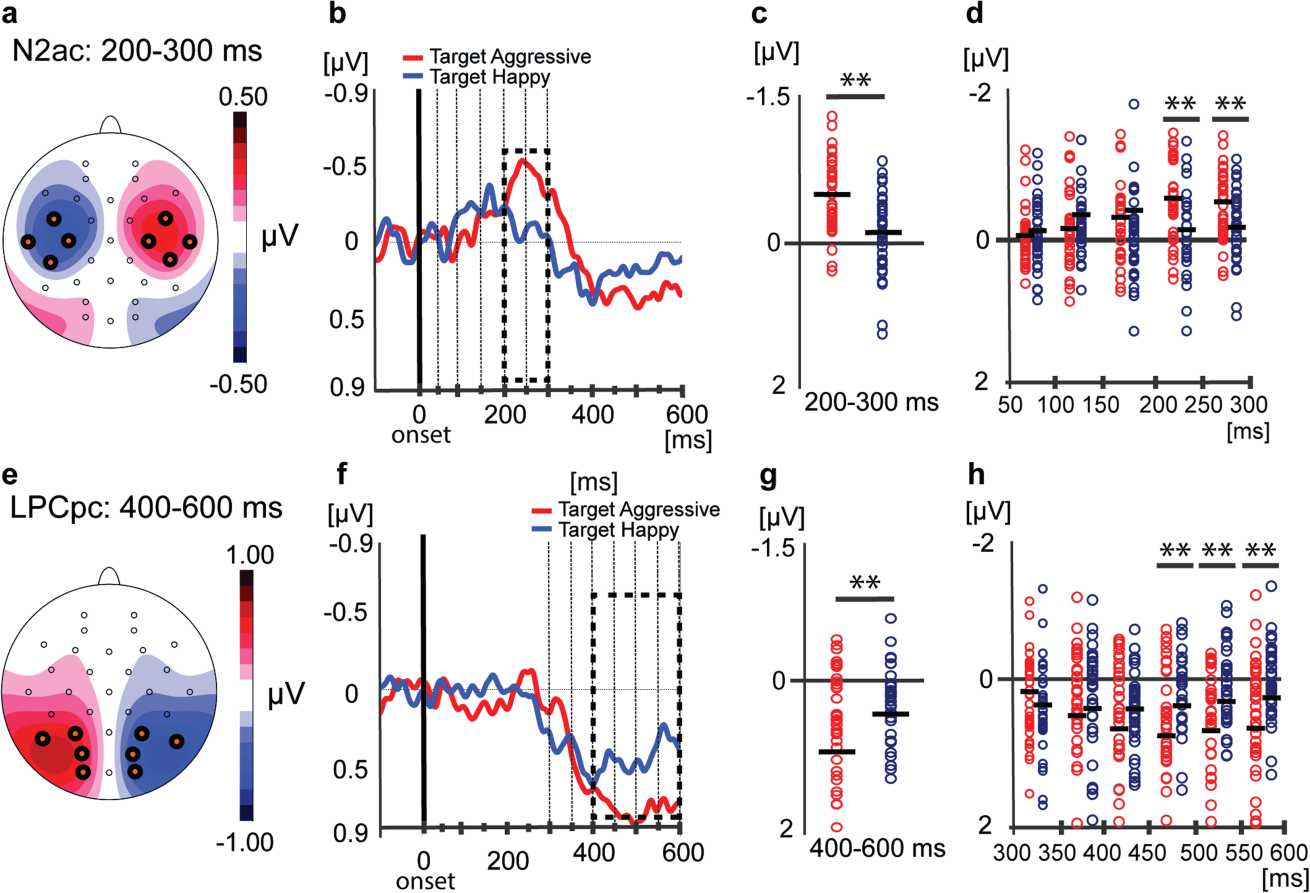
Analyses of the N2ac and LPCpc are shown in the top and bottom panels, respectively. Topographies of the N2ac and LPCpc with clusters of electrodes of interest are shown in the left panels (N2ac in **A**, left hemisphere: C3, CP5, FC5 and T7; right hemisphere: C4, CP6, FC6 and T8; LPCpc in **E**, left hemisphere: O1, P7, PO3 and P3; right hemisphere: O2, P8, PO4 and P4). (**B** and **F**) Grand average of contralateral minus ipsilateral waveforms within the respective clusters of electrodes for aggressive (red) and happy (blue) voices. The analysis interval is indicated by dashed lines. (**C** and **G**) Individual and group means are indicated by circles and thick horizontal lines, respectively. The means represent the mean voltage difference in the interval indicated by the thick broken lines in (B) and (F). (**D** and **H**) Individual and group means for analysis intervals of 50 ms indicated by the thin dashed lines in (B) and (F). Significant paired *t*-tests are indicated by ^**^ (*P* < 0.01).

To track the temporal dynamics of the attentional processing of vocal signals, we analyzed the ERPs in windows of 50 ms (covering the time from 50 to 300 ms after stimulus onset for the N2ac and from 300 to 600 ms for the LPCpc) to reveal when aggressive and happy voices were significantly different (Figure 2B and F).

Finally, we analyzed non-lateralized auditory and attentional ERPs. First, the auditory N1 component at Cz, where it was maximal from 110 to 160 ms and the auditory P3 at Pz, where it was maximal from 300 to 500 ms (see Supplementary Fig. 2). The N1 and P3 components are reliable indices of low- (Hyde, 1997) and high-level dissimilarities (Polich, 2007) between neutral, happy and aggressive acoustic stimuli. All analyses of mean amplitude were performed using the Statistical Package for the Social Sciences (SPSS 23, Inc., Chicago, IL).

Because no lateralized ERP could be calculated in the neutral condition, only means from target-present trials (aggressive and happy) were entered into the repeated measures analysis of variance (ANOVA) on the N2ac and the LPCpc. For the non-lateralized N1 and P300, a repeated-measure ANOVA was conducted on all three conditions (aggressive, happy and neutral).

## Results

### Anxiety level

The overall mean STAI-S score was 32 (s.d. = 6) and the mean STAI-T score was 50 (s.d. = 4).

### Stimulus evaluation

We performed a repeated-measure ANOVA on valence and intensity measures with emotional expression (neutral, aggressive and happy) as factor. As expected, participants rated aggressive vocal signals as more negative (−31.06) than neutral (−0.8) and happy voices [(28.7), *F*(2, 64) = 398.28, *P* < 0.001, partial η^2^ = 0.91], showing more negative emotional valence for aggressive as compared to happy, [*t*(33) = 21.89, *P* < 0.001] and neutral voices [*t*(33) = 19.62, *P* < 0.001]. Happy and neutral voices differed as well, *t*(33) = 16.62, *P* < 0.001.

For intensity, the main effect of emotional expression was significant, *F*(2, 34) = 127.22, *P* < 0.001, partial η^2^ = 0.75, showing higher emotional intensity for aggressive (57.36) as compared to happy (46.21), *t*(33) = 4, *P* < 0.001 and neutral voices (12.46), *t*(33) = 15, 89, *P* < 0.001. Happy and neutral voices differed as well, *t*(33) = 10.71, *P* < 0.001.

### Reaction times

We found a main effect of emotional expression, *F*(2, 25278) = 316.78, *P* < 0.001. Compared to neutral (target-absent) trials, participants responded faster to aggressive, *b* = −0.07, SE = 0.0036, *P* < 0.001; and happy voices, *b* = −0.085, *SE* = 0.0038, *P* < 0.001. Additionally, responses were slower to aggressive as compared to happy voices, *b* = 0.014, *SE* = 0.0043, *P =* 0.001. No effect of gender was found (see Supplementary data).

### Lateralized ERPs

The analysis of data normality is reported in the Supplementary data.

#### 

***Topography.*** Figure 2A and E shows that the N2ac emerges over anterior sites and the LPCpc over parietal sites, consistent with the previous literature (Gamble and Luck, 2011; Gamble and Woldorff, 2015; Lewald *et al.*, 2016).

#### N2ac (200–300 ms)

As shown in Figure 2B and C, the N2ac was modulated by emotional expression with a larger amplitude for aggressive (−0.39 μV) than happy voices (−0.07 μV), *F*(1, 32) = 11.62, *P* = 0.002, partial η^2^ = 0.26. The N2ac was different from zero for aggressive, *t*(33) = 5.44, *P* < 0.001, but not happy voices, *t*(33) = 1.0, *P* = 0.32. The effect of emotion interacted with the gender, *F*(1, 32) = 4.46, *P* = 0.042, partial η^2^ = 0.12. The difference between aggressive (−0.47 μV) and happy (−0.04 μV) was significant for female, *F*(1, 22) = 13.35, *P* < 0.001, partial η^2^ = 0.37, but not for male participants (−0.21 *vs* −0.16 μV), *F*(1, 10) = 0.35, *P* = 0.57, partial η^2^ = 0.03.

#### LPCpc (400–600 ms)

As shown in Figure 2F and G, the LPCpc was larger for aggressive (0.72 μV) as compared to happy voices (0.45 μV), *F*(1, 32) = 9.42, *P* = 0.004, partial η^2^ = 0.22. The mean LPCpc was larger than zero for both aggressive [*t*(33) = 7.54, *P* < 0.001] and happy voices [*t*(33) = 5.9, *P* < 0.001]. No interaction with gender was found (*P* = 0.33).

#### HEOG

The HEOG was not affected by emotion between 200 and 300 ms, *P* = 0.20 or 400–600 ms, *P* = 0.55 (see Supplementary Fig. 2).

#### Time course

To assess the time course, we divided the periods of interest (N2ac, 50–300 ms; LPCpc, 300–600 ms) further into 50 ms time windows. As shown in Figure 2D, we found a main effect of time window on the N2ac, *F*(3.12, 99.85) = 5.670, *P* < 0.001, partial η^2^ = 0.15, showing that the N2ac increased over time. More interestingly, time window and emotional expression interacted, *F*(3.2, 102.44) = 10.89, *P* < 0.001, partial η^2^ = 0.25, reflecting significant differences between aggressive and happy voices from 200 to 250 and 250 to 300 ms (differences of −0.33 and −0.31 μV, respectively), *ts*(33) > 3.2, *P* < 0.002, which were not observed in earlier time intervals. Further, we note that the N2ac collapsed across aggressive and happy voices was significantly different from zero as early as 100–150 ms (−0.14 μV), *t*(33) = 2.68, *P* = 0.011 and 150–200 ms (−0.20 μV), *t*(33) = 3.41, *P* = 0.002. Including gender revealed an interaction by time window, *F*(4,128) = 3.27, *P* < 0.022, partial η^2^ = 0.093, showing a women-specific effect between 200 and 300 ms for the N2ac (see Supplementary data).

As shown in Figure 2H, we found a main effect of time window on the LPCpc, *F*(2.71, 87.013) = 9.19, *P* < 0.001, partial η^2^ = 0.22, showing that the LPCpc increased over time. More interestingly, time window and emotional expression interacted, *F*(3.87, 123.97) = 8.14, *P* < 0.001, partial η^2^ = 0.2, showing that aggressive and happy voices differed between 450 and 500, 500 and 550 and 550 and 600 ms, as evidenced by paired *t*-tests, *t*s(33) > 2.86, *P <* 0.007, but not before 400 ms (*Ps* > .20). From 400 to 450 ms, the difference between aggressive and happy approached significance, *P* = 0.08. Collapsed across emotion, the LPCpc was significantly different from zero as early as 300–350 ms (0.25 μV), *t*(33) = 3.48, *P* = 0.001 and 350–400 ms (0.51 μV), *t*(33) = 6.68, *P* < 0.001. Including gender did not reveal any further effects (all *P* > 0.35).

### Non-lateralized ERPs

#### N1

An ANOVA with emotional expression (neutral, aggressive and happy) as a repeated measures factor on the mean voltage in the interval from 110 to 160 ms was non-significant (−3.04, −2.9, −2.9 μV), *F*(1.62, 51.96) = 0.12, *P* = 0.88. The effect of emotion did not interact with the gender, *P* = 0.84.

#### P300

A similar ANOVA between 300 and 600 ms revealed a significant effect of emotional expression, *F*(2, 64) = 66.8, *P* < 0.001, partial η^2^ = 0.67. The P300 was larger for aggressive (4.59 μV) and happy (3.68 μV) voices as compared with neutral voices (1.23 μV), *t*(33) > 7.9, *P* < 0.001. Additionally, the P300 was larger for aggressive as compared to happy voices, *t*(33) = 3.19, *P* = 0.008. The amplitude of the P300 interacted with gender, *F*(1, 64) = 3.98, *P* = 0.023, partial η^2^ = 0.11 (see Supplementary data).

## Discussion

We examined behavioral and electrophysiological measures of attentional deployment toward threatening voices. Contrary to predictions, aggressive voices were detected more slowly than happy voices. In line with our predictions, the electrophysiological results showed that aggressive voices resulted in a larger amplitude of the lateralized N2ac and LPCpc components. This effect was present in women but not in men for the N2ac. Further, non-lateralized components were consistent with the previous literature. Aggressive and happy voices were not different at an early stage of auditory processing (N1; cf. Liu *et al.*, 2012), whereas differences emerged at a later stage. The larger P3 component to aggressive voices as compared to happy voices suggests that attention to threatening stimuli was enhanced, as reported in previous work in the visual modality (for instance, Delplanque *et al.*, 2006). Thus, threat-related human vocal signals influence processing not only at an early stage but also at a later stage associated with the extraction of emotional meaning (Pell *et al.*, 2015).

The finding of slower responses to aggressive compared to happy voices is puzzling because the threat-capture hypothesis claims that threatening stimuli are given attentional priority. In addition, our electrophysiological indices of attention were enhanced for threatening voices, which we expected to result in faster responses. To understand the cause of this counter-intuitive result, we ran an additional experiment that is presented in the Supplementary data. When emotional or neutral voices were presented without a neutral distractor (i.e. unilaterally), responses were faster to aggressive than to happy voices, which is in line with the threat-capture hypothesis. While we do not have a conclusive interpretation, we think that it is likely that post-attentional processes explain the slower RTs to aggressive voices. Perhaps it was more difficult to discriminate aggressive from neutral voices, which increased the time needed to take a decision about target presence. Decision processes are reflected in RTs but may succeed the early attentional stages that were reflected in the lateralized ERPs. However, we admit that more research is needed to clarify this issue.

Our EEG results provide the first evidence of the early and enhanced deployment of spatial attention toward aggressive voices. Our results are in line with the growing body of evidence showing that rapid emotional salience detection occurs within the first 200 ms after the onset of a non-lateralized voice for explicit processing of emotional voices (e.g. Paulmann and Kotz, 2008; Sauter and Eimer, 2010; Liu *et al.*, 2012; Pinheiro *et al.*, 2013; Schirmer *et al.*, 2013; Pell *et al.*, 2015). Previous research tackling the temporal dynamics of vocal emotional processing used non-lateralized stimuli or indirect measures of attention (Brosch *et al.*, 2008a; Brosch *et al.*, 2009), inherently neglecting that auditory attention is *spatially* oriented toward threatening voices (Öhman and Mineka, 2001). Enhanced spatial orienting in the auditory modality is consistent with voluntary or involuntary orientation toward threatening faces in the visual modality (i.e. Burra *et al.*, 2016; Feldmann-Wustefeld *et al.*, 2011).

The subsequent modulation of the LPCpc component complements the N2ac results. In Gamble and Luck’s study (2011), participants were required to detect the presence or absence of a specific auditory target, this late positivity was associated with attentional reorienting from the attended location back to the fixation. Because the LPCpc was larger for aggressive than happy voices in the present study, we conclude that attentional reorienting was stronger for aggressive voices. From a functional point of view, reorienting to central fixation may optimize detection of threat coming from unpredictable locations in the environment. That is, staying focused on a lateral position may lead to difficulties in detecting threatening stimuli on the opposite side whereas a central focus ensures a balanced intake of sensory information. Alternatively, the LPCpc could underlie disengagement from the target and reorienting to the distracting stimulus to allow for verification of the initial evaluation of the stimulus. In fact, similar to the LPCpc in several respects, the auditory-evoked contralateral occipital positivity (ACOP) has been uncovered for task irrelevant unilateral auditory stimuli (McDonald *et al.*, 2013; Feng *et al.*, 2014). The ACOP has been interpreted as a lateralized neural activity in the visual cortex triggered by the involuntary orienting of visual attention to a non-predictive sound location, which might fit with multimodal results of Brosch *et al.* (2008a, 2009). Overall, it is clear that more research is necessary to understand the function of the LPCpc.

The electrophysiological effect of gender on N2ac ERPs is consistent with prior studies showing that female participants were more sensitive to vocal emotions than male participants when vocal emotions were task-irrelevant (for instance Schirmer *et al.*, 2002; Schirmer *et al.*, 2004; Schirmer *et al.*, 2005; Schirmer *et al.*, 2013). However, the current study was not designed to address gender differences, which explains why there were fewer male than female participants (*N*_male_ = 13 *vs N*_female_ = 21). Thus, the conclusion that the effect of threat on the N2ac only occurred in women is limited by the lower statistical power in the group of men, in addition to the fact that no effect of gender was found for the behavioral results. Nevertheless, in light of the previous literature on this topic, it seems likely that the neural correlates of the early attentional deployment to aggressive vocal signals differ between male and female participants.

Another limitation of our study concerns the acoustic stimuli. Low-level auditory differences have been controlled for as much as possible to avoid alternative accounts of the larger N2ac for aggressive as compared to happy vocal signals. However, the control of auditory stimulus properties may have induced differences in judged emotional valence and intensity of the material, for instance by reducing the subjective intensity of happy vocal signals. Thus, we cannot entirely rule out confounding effects of perceived emotional intensity, similar to a previous study using controlled visual schematic facial expressions (Burra *et al.*, 2016).

Overall, our results addressed the relationship between attention and aggressiveness in the human central nervous system. The larger amplitude of the N2ac for aggressive as compared with happy voices points to attentional enhancement of threatening stimuli at an early stage of spatial processing. Our electrophysiological results therefore support the proposition of differential attention allocation to threat-relevant and threat-irrelevant vocal signals (Grandjean *et al.*, 2005; Brosch *et al.*, 2008a; Brosch et al., 2009). In the visual modality, it was assumed that a feature map represents threat at a pre-attentive stage (Hansen and Hansen, 1988). Our results extend this possibility to the auditory domain. Taken together, our results speak in favor of early differences in attentional orienting as suggested by the ‘threat-capture’ hypothesis (LeDoux, 1996; Öhman and Mineka, 2001), thereby extending the scope of the hypothesis beyond the visual modality. In fact, it is plausible that subcortical processes (‘low road’) would determine the preferential orienting response to threat, as operationalized by a larger amplitude of the N2ac. In contrast, the LPCpc could be the consequence of attentional reorienting following disengagement from the target. Disengagement is likely influenced by top-down goals, because potential threats are eventually also cognitively evaluated after attentional selection (‘high road’). Reorienting of attention may also play a crucial role when voices compete in space although this mechanism has so far been neglected in the literature. In the case of auditory attention, orienting could at least partly rely on the amygdala or the superior temporal gyrus/sulcus while the region underlying attentional disengagement could be the prefrontal/orbitofrontal cortex (Grandjean *et al.*, 2005; Sander *et al.*, 2005; Ceravolo *et al.*, 2016). The complementary roles of attentional orienting, reorienting and/or disengagement and their neural correlates should therefore be the subject of future research because of their relevance for affective neuroscience.

## Conclusions

Measurements of the N2ac and LPCpc components suggest different attentional selectivity for threatening and happy voices. Our results extend conclusions from the visual modality and reveal that the rapid orienting/engagement toward threatening stimuli as well as the rapid reorienting/disengagement from threatening stimuli are fundamental neural mechanisms occurring both in the visual and auditory modality. In sum, our results reveal a general, dynamic principle for the organization of the relationship between spatial attention and threat detection in the human central nervous system.

## Supplementary Material

Supplementary_data_r4
