# Supplementary material for: Early spatial attention deployment toward and away from aggressive voices
Source: Soc Cogn Affect Neurosci. 2018 Nov 9;14(1):73–80. doi: 10.1093/scan/nsy100 (PMC6318470; doi:10.1093/scan/nsy100)
Supplement: Supplementary_data_r4 [file supplementary_data_r4.docx]

**SUPPLEMANTARY MATERIAL**

**Supplementary Experiment with Unilateral Stimulation**

We ran an experiment in which participants were required to detect the presence or the absence of a single emotional vocal signal. Based on the “threat-capture hypothesis” (Öhman & Mineka, 2001), we predicted faster detection responses with aggressive compared to happy targets. Stimuli were presented unilaterally, without neutral distractor.

**Materials and Method**

**Participants**

There were 17 healthy participants (6 male, mean age: 25.88 ± 3.9 years, ranging from 20 to 33 years). All participants reported normal or corrected-to-normal vision, normal hearing and no neurological or psychiatric problems. Informed consent was obtained from all participants before testing. The experiment was approved by the Institutional Review Board (Ethics Committee) of the University of Geneva.

**Stimuli**

We used the same stimuli as in the EEG experiment.

**Procedure**

The procedure was similar to the EEG experiment, except that sounds were presented unilaterally. Participants were requested to detect the presence of an emotional target (aggressive or happy). Target-present and target-absent trials were equally likely. On target-absent trials, a neutral vocal sound was played. The stimulus was equally likely to be played by the left and right loudspeaker. In order to control for the potential effects of target predictability (Burra & Kerzel, 2013), the target category (aggressive or happy) was fixed during 64 trials in the blocked version of the task. In the mixed version, the target category alternated randomly. The experiment consisted of 6 blocks of 64 trials subdivided into 2 mini-blocks of 32 trials, with a rest period of minimum 10 s. The order of aggressive and happy was counterbalanced across participants, but was always followed by a mixed block. Shortly before the experiment, all stimuli were shown once to familiarize participants with the stimuli.

**Results**

The analyses were similar to the EEG experiment. For the sake of clarity, we reported the raw data in millisecond in the Supplementary Table 3 even though the log of the reaction times was used in the analysis.

**Response time**

We found a main effect of emotional expression, *F*(2, 3559.1) = 38.75, *p* < .001, but no effect of gender, *p =* .225, and no interaction, *p* > .50. Participants were faster at detecting aggressive compared to neutral, *b* = -0.096, *SE* = 0.0024, *p* <.0001, and happy compared to neutral voices, *b* = -0.050, *SE* = 0.0030, *p* < .0001. Importantly, they were also faster to detect aggressive than happy voices, *b* = -0.046, *SE* = .0028, *p* = .0003, and generally faster for emotional (aggressive & happy) as compared to neutral voices, *b* = -0.146, *SE* = .0012, *p* < .0001.

**Accuracy**

No significant difference was observed, *p*s > .25.

**Discussion**

Participants were faster to detect the presence of agressive compared to happy or neutral voices. These results are consistent with the anger superiority effect (see also Eastwood, Smilek, & Merikle, 2001; Fox et al., 2000; Hansen & Hansen, 1988; Lundqvist, Esteves, & Ohman, 1999; Tipples, Atkinson, & Young, 2002). Thus, our stimuli capture the enhanced sensitivity to threat-related compared to neutral stimuli that has originally been discovered for visual stimuli (Öhman, Flykt, & Esteves, 2001).

**Supplementary Methods and Results**

**Materials and Method**

**Control of low-level acoustic features**

Pitch, duration, and intensity of each stimulus were measured using the Praat software (Boersma & Weenink, 2016). Analysis of the original stimuli revealed small but significant differences between emotions in pitch level and intensity (see Supplementary Table 1). To balance the stimuli, we slightly altered duration, pitch and then intensity of some happy and neutral stimuli, using a shareware version of GoldWave digital audio editor. Aggressive stimuli remained unchanged. After adjustment, the differences between emotions disappeared (see Supplementary Table 2).

**Evaluation of the threatening content of the voice stimuli**

Twenty-nine participants who had not participated in the other experiments rated all experimental stimuli, including the stimulus we removed. In this rating, participants assessed the voices according to four categories (neutral, happy, angry and sadness) as well as their threatening content on a continuous scale (0 = non-threatening to 100 = highly threatening). Analysis of the rating results revealed that stimuli were well categorized (mean accuracy for aggressive voices: 81.90%, *SD* = 12.31; mean accuracy for happy voices: 81.91%, *SD* = 14.01). Aggressive voices were rated as more threatening (43) than happy voices (6), *t*(28) = 15.06, *p* < .001, and neutral (4), *t*(28) = 13.42, *p* < .001, while happy and neutral voices were not different, *t*(28) = 1.36, *p* = .18.

**Results**

**Effects of stimulus order (blocked vs. random)**

**Anxiety level.** The mean STAI-S scores were 33 (*SD* = 6.3) and 30 (*SD* = 9.6) for each version of the task (blocked vs. random), respectively. The mean STAI-T scores were 50 (*SD* = 4.6) with blocked and 50 (*SD* = 2.3) with pseudo-random presentation. Two independent t-tests performed on both scores did not reveal any differences as a function of stimulus order, all *p* > .41.

**N2ac (200-300 ms)**. Including presentation order as a between-subjects factor in the ANOVA reported in the main text revealed a main effect of presentation order, *F*(1, 32) = 8.11 *p* = .008, partial η2 = 0.2, showing a larger N2ac for blocked (-0.38 µV) than random (0.08 µV) order of presentation. Critically, emotional expression and presentation order did not interact, *p* = .709.

**LPCpc (400-600 ms)**. Including presentation order did not produce any further effects, *p*s > .14.

**N2ac (time course).** We confirmed the effect of presentation order, *F*(1, 32) = 7.78, *p* = .009, partial η2 = 0.19, that was already observed in the analysis of the 200-300 ms interval. The N2ac was larger for blocked (-0.28 µV) as compared to a random (-0.06 µV) order. Emotional expression and presentation order did not interact, *p* = .69.

**LPCpc (time course)**. Including presentation order did not reveal any additional effects, *p*s > .14.

**N1 and P300.** Including presentation order did not reveal any further effects, *p*s > .22.

**Effects of gender**

There were 13 male and 21 female participants in the sample.

**Reaction time.** Including gender in our GLM model did not yield an effect of gender, *p* = .199, or an interaction between gender and emotion, *p* = .642.

**N2ac**.The effect of emotion interacted with the gender, *F*(1, 32) = 4.46, *p* = .042, partial η^2^ = .12. The difference between aggressive (-0.47 µV) and happy (-0.037 µV) was significant for female, *F*(1, 22) = 13.35, *p* < .001, partial η^2^ = .37, but not for male participants (-0.21 vs. -0.16 µV), *F*(1, 10) = .35, *p* = .57, partial η^2^ = .03.

**LPCpc**. The effect of emotion did not interact with the gender, p = .33.

**Time-course: N2ac.** Including gender revealed an interaction by time window, *F(4,128) = 3.27, p < .022*, partial η^2^ = .093. Post-hoc analysis highlighted that while this difference between aggressive and happy prosody is significant between 200-250 and 250-300ms in female, *ts*(22) > 3.26, *ps* < .004, but not before (*ps* < .14), this difference did not reach significance in male participants throughout all the time windows, *ps* < .29,

**Time-course: LPCP.** Including gender did not reveal any further effects, *p*s > .35.

**N1**. The effect of emotion did not interact with the gender, p = .84.

**P300.** The amplitude of the P300 interacted with gender, *F*(1, 64) = 3.98, *p* = .023, partial η^2^ = .11. Post-hoc independent samples t-test revealed that for aggressive voices, the P300 was larger for female (5.42 µV) than male participants (2.86 µV), *t*(31.97) = 2.53, *p* = .017. Differences between female and male participants did not reach the level of significance for neutral or happy conditions, *p* = .42 and .12, respectively.

**Data normality**

The N2ac amplitudes between 200-300 ms for aggressive and happy conditions did not significantly deviate from normality, *p* = .85 and *p* = .59, respectively. Levene’s test of equality of error variances revealed that the variances were homogenous, all *p*s > .12.

Further, N2ac amplitudes for 50 ms analysis windows in the time course analysis did not significantly deviate from normality, *p*s > .15, except for the 50-100 ms time window with aggressive voices, *p* = .001, Kolmogorov-Smirnov (34) = .172. Levene's test of equality of error variances revealed that the variances were homogenous, *p*s > .07. One condition, the happy condition between 150-200 ms did not fulfill this prerequisite.

The LPCpc amplitudes for 50 ms analysis windows in the time course analysis did not significantly deviate from normality, *p*s > .73, except for the 350-400 ms time window with happy voices, *p* = .04, Kolmogorov-Smirnov (34) = .164. Levene's test of equality of error variances revealed that the variances were homogenous among conditions, *p*s > .07.**Supplementary References**

Boersma, P., & Weenink, D. (2016). Praat software. University of Amsterdam. *Im Internet: <http://www>. fon. hum. uva. nl/praat*.

Burra, N., & Kerzel, D. (2013). Attentional capture during visual search is attenuated by target predictability: evidence from the N2pc, Pd, and topographic segmentation. *Psychophysiology, 50*(5), 422-430. doi:10.1111/psyp.12019

Eastwood, J. D., Smilek, D., & Merikle, P. M. (2001). Differential attentional guidance by unattended faces expressing positive and negative emotion. *Percept Psychophys, 63*(6), 1004-1013.

Fox, E., Lester, V., Russo, R., Bowles, R. J., Pichler, A., & Dutton, K. (2000). Facial Expressions of Emotion: Are Angry Faces Detected More Efficiently? *Cogn Emot, 14*(1), 61-92. doi:10.1080/026999300378996

Hansen, C. H., & Hansen, R. D. (1988). Finding the face in the crowd: an anger superiority effect. *J Pers Soc Psychol, 54*(6), 917-924.

Lundqvist, D., Esteves, F., & Ohman, A. (1999). The face of wrath: Critical features for conveying facial threat. *Cognition & Emotion, 13*(6), 691-711. doi:Doi 10.1080/026999399379041

Öhman, A., Flykt, A., & Esteves, F. (2001). Emotion drives attention: detecting the snake in the grass. *J Exp Psychol Gen, 130*(3), 466-478.

Öhman, A., & Mineka, S. (2001). Fears, phobias, and preparedness: toward an evolved module of fear and fear learning. *Psychol Rev, 108*(3), 483-522.

Tipples, J., Atkinson, A. P., & Young, A. W. (2002). The eyebrow frown: a salient social signal. *Emotion, 2*(3), 288-296.

**Supplementary Table 1.** Analysis of acoustic properties of the 24 experimental stimuli (8 per conditions) prior to adjustment. Mean and standard deviation of acoustical parameters for neutral, aggressive and happy vocal signals are shown. Separate one-way ANOVAs on pitch, duration and intensity found significant differences between the three conditions for pitch and a tendency for duration. Therefore, the original stimuli from Bänziger and Scherer (2007) were adjusted (see Supplementary Table 2).

|  | Pitch (Hz) | | Duration (ms) | Intensity (dB) |
| --- | --- | --- | --- | --- |
| Neutral (n = 8) | 219 ± 101 | | 597± 6 | 81.4 ± 2.4 |
| Aggressive (n = 8) | 288 ± 94 | | 597 ± 11 | 79.6 ± 9.6 |
| Happy (n = 8) | 333 ± 47 | | 581 ± 24 | 77 ± 3.2 |
| *F*(2, 21) | | 3.69 | 2.96 | 1.1 |
| *p* | | .042 | .07 | .34 |

Hz = Hertz, ms = milliseconds and dB = decibels.

**Supplementary Table 2.** Analysis of acoustic properties of the 24 experimental stimuli (8 per conditions) after adjustment. Separate one-way ANOVA on pitch, duration and intensity no longer revealed differences between the three conditions.

|  | Pitch (Hz) | | Duration (ms) | Intensity (dB) |
| --- | --- | --- | --- | --- |
| Neutral (n = 8) | 246 ± 69 | | 548 ± 97 | 81.3 ± 2.62 |
| Aggressive (n = 8) | 288 ± 94 | | 597 ± 11 | 79.6 ± 9.6 |
| Happy (n = 8) | 300 ± 58 | | 596 ± 59 | 80.59 ± 1.6 |
| *F*(2, 21) | | 1.12 | 1.41 | .18 |
| *p* | | .34 | .26 | .83 |

Hz = Hertz, ms = milliseconds and dB = decibels.

**Supplementary Table 3**. Mean reaction time and accuracy with standard error (in parenthesis) for unilateral and bilateral experiments. The bilateral experiment was reported in the main text, the unilateral experiment was only reported in the supplementary materials.

|  |  | Target-absent | Target-present Aggressive | Target-present Happy |
| --- | --- | --- | --- | --- |
| Reaction Time | unilateral (N = 17) | 755 (27) | 684 (26) | 721 (26) |
|  | bilateral (N = 34) | 657 (18) | 618 (17) | 606 (18) |
| Accuracy | unilateral (N = 17) | 93.8% (0.7) | 94.3% (0.7) | 93.4% (134) |
|  | bilateral (N = 34) | 94.9% (0.4) | 94.9% (0.5) | 95.1% (0.5) |


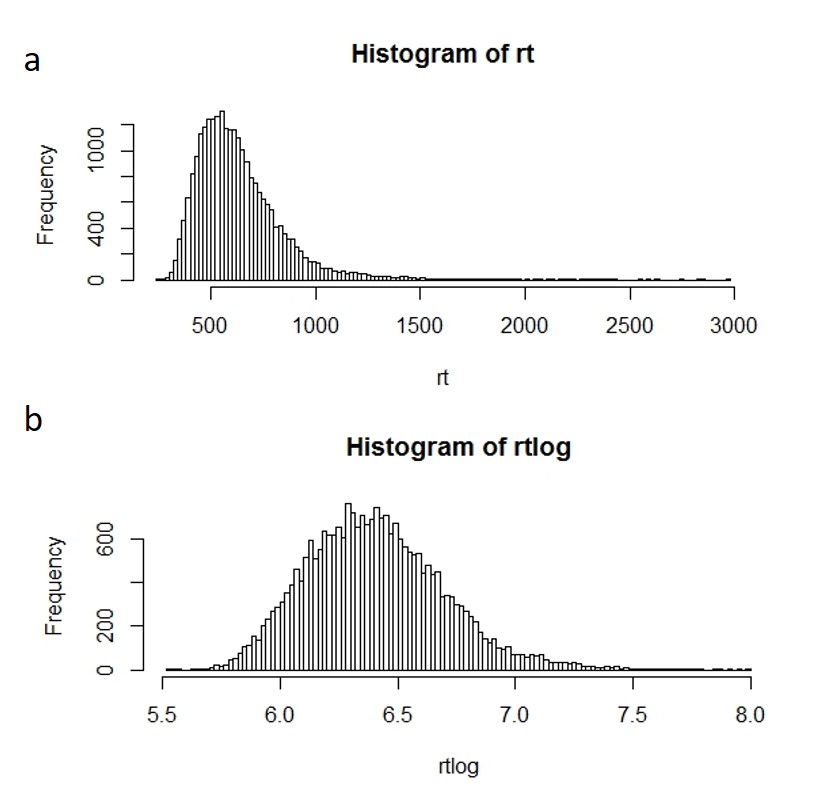


**Supplementary Figure 1.** Illustration of behavioral data distribution before (a) and after applying a log transform (b). The improvement from panel (a) to panel (b) can be clearly seen and shows the positive impact of the log transform on data normality. *
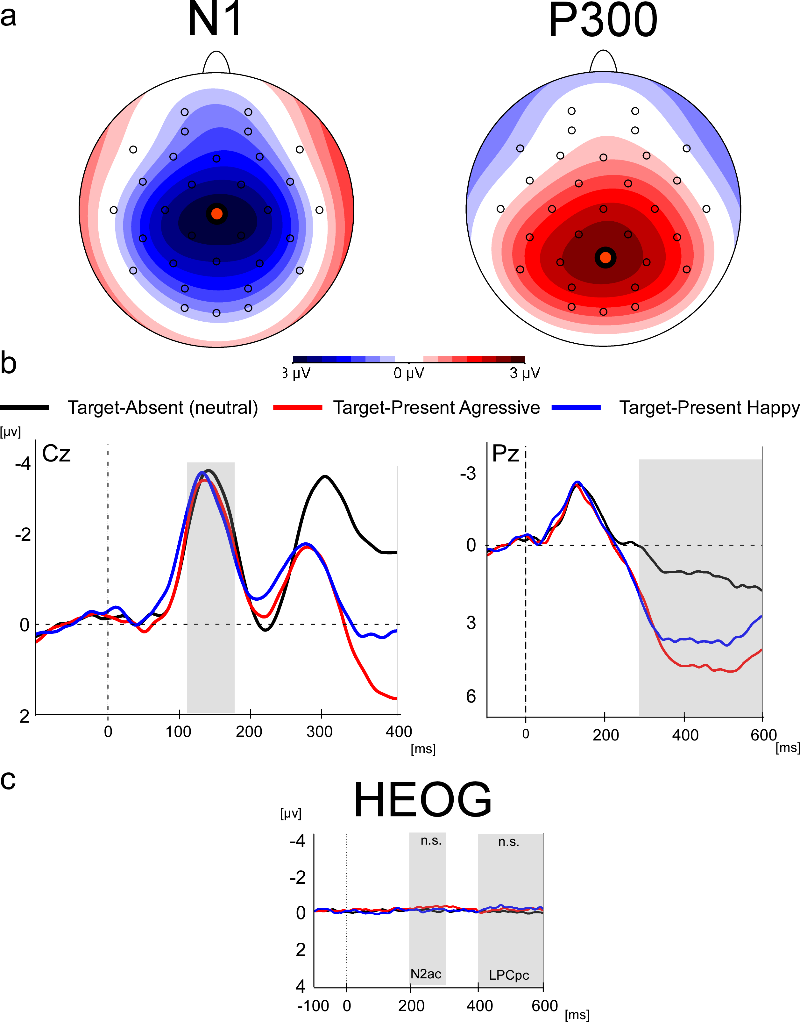
*

**Supplementary Figure 2.** The top panel (a) shows the scalp topography of the difference between target-absent and target presence for the N1 and the P300 components. Consistent with the literature, the N1 was maximal at Cz and the P300 at Pz (see orange circles). The middle panel (b) shows the non-lateralized ERPs at electrodes Cz and Pz. The respective time windows for analysis are shown in gray (110-160 ms for the N1 and 300-600 ms for the P300). The bottom panel (c) shows the horizontal electro-oculogram (HEOG) difference waves. Negative deflections indicate eye movements toward the target. There were no significant difference during the N2ac and the LPCpc time windows (in gray), ruling out that eye movements contaminated the results.
